# Pyogenic Granuloma of the Dorsum of the Tongue in a Child: A Case Report and Literature Review

**DOI:** 10.1155/crpe/6872764

**Published:** 2026-09-28

**Authors:** Elisa M. R. B. Coelho, Júlia Papke, Fabio L. D. M. Maito, Ana Caroline B. Silva, Amanda B. S. Heck, Paulo F. Kramer

**Affiliations:** ^1^ Department of Pediatric Dentistry, Pontifical Catholic University of Rio Grande do Sul (PUCRS), Porto Alegre, Brazil, pucrs.br; ^2^ Department of Oral Pathology, Pontifical Catholic University of Rio Grande do Sul (PUCRS), Porto Alegre, Brazil, pucrs.br; ^3^ Department of Stomatology, Pontifical Catholic University of Rio Grande do Sul (PUCRS), Porto Alegre, Brazil, pucrs.br

**Keywords:** biopsy, child, granuloma, pyogenic, tongue

## Abstract

Pyogenic granuloma (PG) is a reactive lesion that most commonly affects the gingiva but may also occur at less frequent intraoral sites, complicating clinical diagnosis, particularly in pediatric patients. This paper reports a case of lingual PG in a seven‐year‐old girl and provides a descriptive review of published pediatric cases. The lesion developed following a reported episode of local trauma to the tongue, although the specific nature of the trauma was unknown, and presented as a painless, ulcerated nodular growth on the left anterior dorsum of the tongue. An excisional biopsy was performed, and the diagnosis was confirmed by histopathological examination. Complete healing was observed, with no recurrence after 3 months of follow‐up. A descriptive literature review was conducted through a structured search of PubMed, Scopus, and Web of Science for published cases of oral PG in children aged 0–12 years. Thirty‐six cases met the eligibility criteria. Most lesions involved the gingiva, while extragingival sites included the tongue, lips, and palate. Conventional surgical excision was the most frequently reported initial treatment. This case and the literature review highlight that PG may occur at atypical intraoral sites in children and requires careful clinical evaluation and histopathological confirmation.

## 1. Introduction

Pyogenic granuloma (PG) is a benign, non‐neoplastic, reactive lesion [1, 2]. It is one of the most common reactive soft‐tissue enlargements [3]. The term PG, however, is an erroneous term, as the lesion is not associated with pus and does not have the histological characteristics of a granuloma [2, 4]. It is also known as a lobular capillary hemangioma, and when it occurs during pregnancy, it is termed pregnancy epulis or *granuloma gravidarum* [5–7].

PG can occur at any age but is more common in young adults and more frequent in females, likely due to hormonal influences on vascular proliferation; it is uncommon in children [2, 8, 9]. PG accounts for approximately 2% of all oral diseases, and its incidence among reactive oral lesions ranges from 25% to 30% [8, 10]. Moreover, it is most frequently found in the gingiva (approximately 75% of the cases) although it is also occasionally found on the lips, palate, and tongue [2, 3, 11].

The pathogenesis of PG is not fully understood, and some authors describe it as a reactive or reparative tumoral process, meaning a tumor resulting from excessive tissue proliferation rather than a true neoplasm [12]. Trauma is believed to be the most common initial event, and several factors have been associated with its development, including local irritation, chronic trauma, poor oral hygiene, hormonal imbalance, and certain medications [5, 7, 10, 11].

Clinically, an exophytic growth with a sessile or pedunculated base, reddish coloration, and occasional ulcerations is observed [8, 13]. Spontaneous bleeding may also occur due to the high vascularity [14]. Size can range from a few millimeters to several centimeters in diameter, rarely exceeding 25 mm [1, 15]. Radiographic abnormalities are not observed although localized alveolar bone rarefaction may occur in some cases [12].

The diagnosis of PG is based on excisional biopsy and histopathological analysis. Considering the clinical characteristics, the differential diagnosis includes other exophytic lesions with a similar appearance, such as peripheral giant cell granuloma, peripheral ossifying fibroma, metastases of malignant tumors, hemangioma, conventional granulation tissue, fistula, inflammatory gingival hyperplasia, Kaposi’s sarcoma, angiosarcoma, non‐Hodgkin’s lymphoma, and cutaneous horn on the lower lip [8, 16].

Treatment consists of surgical removal of the lesion, combined with elimination of irritant etiological factors, resulting in a low recurrence rate [5, 11]. Extragingival locations are rare, especially in children. This article reviews existing literature and presents a clinical case of PG located at a nongingival site in a child.

## 2. Literature Review

A structured literature search was conducted in PubMed, Scopus, and Web of Science using combinations of the terms “pyogenic granuloma” OR “lobular capillary hemangioma”; child∗ OR infant∗ OR pediatric OR paediatric; oral OR mouth OR gingiva∗ OR buccal OR palate OR lip OR tongue; and “case report” OR “case series” OR “clinical case” OR “report of a case.” The search included clinical case reports and case series of oral PG in children aged 0–12 years, published in English, with no restriction on publication year. Studies that did not address PG, did not involve children within the defined age range, did not report an oral lesion, or were not clinical case reports were excluded. The last search was conducted in December 2025. Study selection was based on title and abstract screening followed by full‐text assessment of potentially eligible reports. Data on patient characteristics, lesion site, diagnostic methods, treatment, follow‐up, and recurrence were manually extracted from the included reports and summarized descriptively. Following screening and eligibility assessment, 36 cases were included in this descriptive literature review. Table 1 summarizes the characteristics of the included cases (Table 1).

**TABLE 1:** Characteristics of case reports included in the review, by year and country of publication, journal, patient profile, lesion location, diagnostic methods, and therapeutic approach.

First author (year)	Country	Journal	Age/sex	Lesion site	Diagnosis	Treatment	Follow‐up	Recurrence
Muench et al. (1992) [17]	USA	Pediatr Dent	6 days Male	Gingiva	Histopathology	Surgical excision	1 month	No

Goodman‐Topper and Bimstein (1994) [18]	Israel	J Dent Child (Chic)	12 years Male	Gingiva	Rx + Histopathology	Surgical excision	2 months	No

Willies‐Jacobo et al. (2000) [19]	USA	Arch Pediatr Adolesc Med	7 months Male	Gingiva	Histopathology	Surgical excision	NR	NR

Akyol et al. (2001) [20]	Turkey	Int J Pediatr Otorhinolaryngol	4 months Male	Tongue	Histopathology	Surgical excision	NR	NR

Sheth et al. (2001) [21]	USA	Arch Pediatr Adolesc Med	1 month Female	Tongue	Histopathology	Surgical excision	1 year	No

Aguillo (2002) [22]	Spain	Int J Paediatr Dent	1 year Male	Gingiva	Rx + Histopathology	Surgical excision	7 months	No

Kneafsey and Hughes (2002) [23]	UK	Dent Update	12 years Female	Tongue	Clinical	Surgical excision	10 days	No

El Mustafa (2003) [24]	Sudan	Saudi Med J	12 years Male	Tongue	Histopathology	Surgical excision	NR	NR

Zielnik‐Jurkiewicz (2005) [25]	Poland	Eur Arch Otorhinolaryngol	2 months Male	Tongue	Histopathology	Surgical excision	3 years	No

Shenoy and Dinkar (2006) [26]	India	J Indian Soc Pedod Prev Dent	8 years Female	Gingiva	Histopathology	Surgical excision	NR	NR

Oliveira et al. (2008) [27]	Brazil	J Dent Child (Chic)	7 years Female	Gingiva	Rx + Histopathology	Surgical excision	1 year	No

Chagas et al. (2009) [14]	Brazil	J Dent Child (Chic)	2 years Female	Upper lip	Histopathology	Surgical excision	1 week	No

Gonçales et al. (2010) [28]	Brazil	J Appl Oral Sci	12 years Female	Upper lip	Histopathology	Surgical excision	4 years	No

Cho et al. (2011) [29]	South Korea	Int J Pediatr Otorhinolaryngol	2 months Male	Palate	Histopathology + MRI	Surgical excision	11 months	No

Deshmukh et al. (2013) [30]	India	Indian J Dent Sci	9 years Male	Gingiva	Histopathology	Surgical excision	1 week	No

Carvalho et al. (2015) [31]	Brazil	J Dent Child (Chic)	11 years Male	Upper lip	Histopathology	Surgical excision	1 year	No

Gokhale et al. (2015) [32]	India	J Clin Diagn Res	9 years Female	Gingiva	Histopathology	Laser surgical excision	6 months	No

Agarwal et al. (2016) [13]	India	J Clin Diagn Res	8 days Male	Gingiva	Histopathology	Surgical excision	3 months	No

Carti et al. (2017) [8]	Turkey	Cumhuriyet Dent J	12 years Female	Gingiva	Rx + Histopathology	Surgical excision	1^st^: 3 months 2^nd^: 1 year	Yes (3 months after 1^st^ treatment)

Peters et al. (2018) [2]	USA	J Clin Pediatr Dent	5 years Female	Tongue	Histopathology	Surgical excision	1 week	No

Costello and Huston (2019) [33]	Ireland	J Paediatr Child Health	12 years Male	Palate	Histopathology	Surgical excision	1 month	No

Tatar et al. (2020) [15]	Romania	Arch Balk Med Union	12 years Male	Lower lip	US + Histopathology	Surgical excision	8 months	No

Cunico et al. (2021) [34]	Brazil	J Clin Pediatr Dent	5 months Female	Gingiva	Histopathology	Laser surgical excision	7 months	No

Mowafy et al. (2021) [35]	Egypt	J Pediatr Surg Case Rep	2 months Male	Palate	Histopathology	Surgical excision	1^st^: 2 months 2^nd^: 18 months	Yes (2 months after 1^st^ treatment)

Pisano et al. (2021) [16]	Italy	Am J Case Rep	11 years Female	Lower lip	US + Histopathology	Laser surgical excision	1 month	No

Rubaian (2021) [36]	Saudi Arabia	Open Access Emerg Med	11 years Female	Lower lip	Clinical	1^st^: Shave excision + electrocautery 2^nd^: Cryotherapy + topical salt	1^st^: 5 days 2^nd^: 2 weeks	Yes (5 days after 1^st^ treatment)

Labedz et al. (2022) [37]	Argentina	Otolaryngol Case Rep	1 month Female	Tongue	MRI + Histopathology	1^st^: Surgical excision 2^nd^: Topical timolol 0.5%	1^st^: 4 weeks 2^nd^: 20 months	Yes (4 weeks after 1^st^ treatment)

Nassar and Besar (2022) [11]	Egypt	Egypt J Otolaryngol Allied Sci	10 years Female	Tongue	Histopathology	Surgical excision using coblation	6 months	No

Tedjosasongko et al. (2022) [38]	Indonesia	J Int Dent Med Res	10 years Male	Upper lip	Histopathology	Electrocautery surgical excision	1 week	No

Jayam (2023) et al. [39]	India	Cureus	3 years Male	Gingiva	Rx + Histopathology	Surgical excision	3 months	No

Vaidya et al. (2023) [9]	Nepal	J South Asian Assoc Pediatric Dent	11 years Female	Gingiva	Rx + Histopathology	Surgical excision	6 months	No

Henry et al. (2024) [40]	France	Pan Afr Med J	11 years Female	Gingiva	Rx + CBCT + Histopathology	1^st^: Surgical excision (6 procedures) 2^nd^: Intralesional + topical corticosteroids	1^st^: 46 months 2^nd^: 18 months	Yes (after 6 surgical excisions)

Vishnu et al. (2024) [6]	India	J Clin Diagn Res	6 years Male	Gingiva	Rx + Histopathology	Electrocautery surgical excision	6 months	No

Goyal et al. (2025) [41]	India	BMJ Case Rep	10 years Male	Gingiva	MRI + Histopathology	Laser surgical excision	1 year	No

Iram et al. (2025) [7]	India	Pediatr Dent J	12 years Female	Gingiva	CBCT + Histopathology	Electrocautery surgical excision	3 months	No

Jana et al. (2025) [10]	India	J Oral Res Rev	12 years Female	Gingiva	Rx + Histopathology	Surgical excision	3 months	No

*Note:* Rx, radiography; US, ultrasound.

Abbreviations: CBCT, cone‐beam computed tomography; MRI, magnetic resonance imaging; NR, not reported.

Patient age ranged from 6 days to 12 years, with a nearly equal distribution between females (*n* = 19; 52.8%) and males (*n* = 17; 47.2%). The lesions were located on the gingiva (*n* = 18; 50.0%), tongue (*n* = 8; 22.2%), lips (*n* = 7; 19.4%), and palate (*n* = 3; 8.3%). Histopathological examination was reported in most cases (*n* = 34; 94.4%), while complementary examinations, including radiography, magnetic resonance imaging (MRI), cone‐beam computed tomography (CBCT), and ultrasound, were used in selected cases. Conventional surgical excision was the most frequently reported initial treatment, whereas alternative approaches included laser excision, electrocautery, coblation, cryotherapy, topical agents, and corticosteroid therapy. Follow‐up and recurrence data, when reported, are summarized in Table 1.

## 3. Case Presentation

This report followed CARE guidelines [42] and was approved by the Human Research Ethics Committee (CAAE: 93998825.1.0000.5336) of the Pontifical Catholic University of Rio Grande do Sul (PUCRS), Porto Alegre, Brazil. Written informed consent for publication was obtained from the patient’s legal guardian.

### 3.1. Patient Information and Clinical Findings

A 7‐year‐old female patient was referred to the Pediatric Dentistry Service for evaluation of a nodular lesion on the left anterior dorsum of the tongue. According to the patient’s guardians, the lesion had developed 2 months earlier following a reported episode of local trauma to the tongue although the specific nature of the trauma was unknown. After the lesion developed, the patient frequently bit the affected area, which may have contributed to continued local irritation.

Extraoral examination revealed no abnormalities. A painless, pedunculated, ulcerated nodule was observed on the left anterior dorsum of the tongue. Based on the clinical findings, a provisional diagnosis of PG was established. The clinical dimensions of the lesion were not recorded before excision.

Differential diagnoses included hemangioma, peripheral giant cell granuloma, inflammatory gingival hyperplasia, fibrous epulis, and Kaposi’s sarcoma; however, these conditions cannot be reliably distinguished based on clinical features alone.

### 3.2. Treatment

The treatment plan consisted of an excisional biopsy followed by histopathological analysis. The procedure was performed under local anesthesia with 2% lidocaine containing a vasoconstrictor. Tongue traction was achieved with a 4‐0 nylon suture, followed by an incision with a No. 15 scalpel blade, complete removal of the lesion, and closure with 4‐0 Vicryl sutures. An analgesic was prescribed postoperatively.

At the 7‐day follow‐up, the patient reported no pain or complications, and the sutures were removed. Complete healing was observed at 30 days, with no ulceration or scarring. At the 3‐month follow‐up, the patient remained asymptomatic and showed no evidence of recurrence (Figure 1).

**FIGURE 1:**
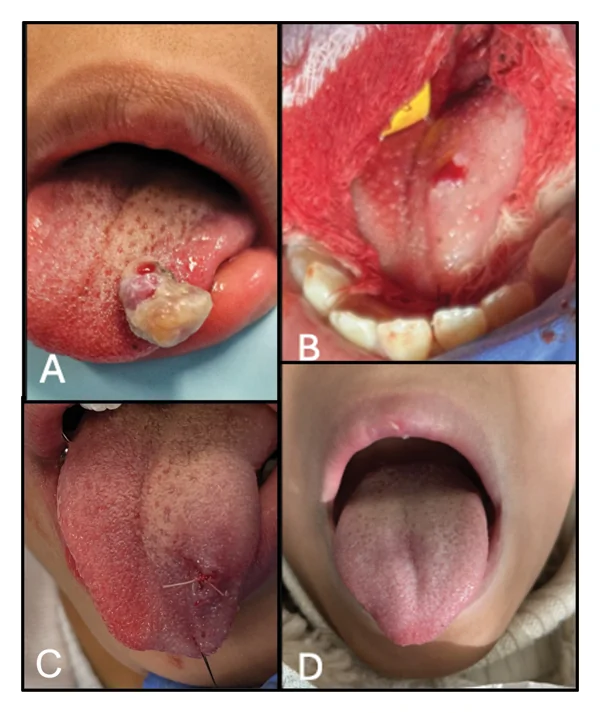
(A) Detail of ulcerated exophytic lesion on the left anterior dorsum of the tongue. (B) Intraoral view after excision of the lesion. (C) Immediate suturing of the operated area. (D) Clinical appearance after 90 days, with complete tissue repair and absence of recurrence.

### 3.3. Histopathological Findings

The excised specimen was fixed in 10% formalin and submitted for histopathological analysis. Macroscopic examination revealed a brownish, irregular, firm soft‐tissue fragment measuring 1.5 × 1 × 1 cm.

Microscopic examination showed stratified squamous epithelium, partially hyperplastic and ulcerated, overlying fibrous connective tissue with a lymphoplasmacytic inflammatory infiltrate, areas of suppuration, and prominent vascular proliferation. These findings confirmed the diagnosis of PG (Figure 2).

**FIGURE 2:**
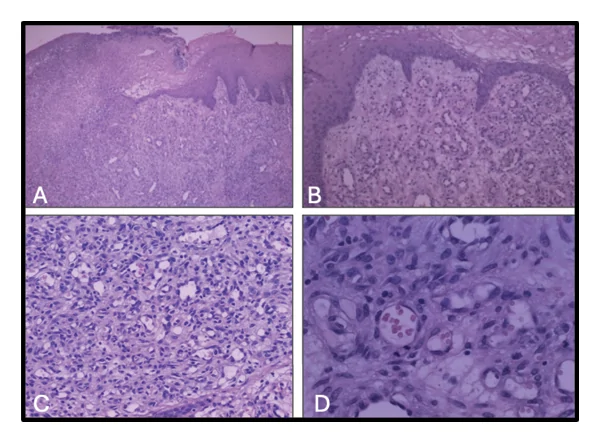
Histopathological features of lingual pyogenic granuloma (hematoxylin and eosin [H&E] stain); (A) H&E, × 40: hyperplastic stratified squamous epithelium with a parakeratinized surface and an ulcerated area covered by a fibrinopurulent membrane overlying fibrous connective tissue with chronic inflammatory infiltrate and vascular neoformation. (B) H&E, × 100: hyperplastic epithelium with hydropic degeneration overlying fibrous connective tissue with newly formed vessels and areas of edema. (C) H&E, × 200: Proliferation of endothelial cells associated with vascular neoformation. (D) H&E, × 400: Vascular neoformation showing plump endothelial cells.

## 4. Discussion

PG is a reactive, non‐neoplastic fibrovascular proliferation commonly associated with chronic local irritation or trauma [4, 8]. Although it predominantly affects the gingiva, the present case highlights an uncommon extragingival presentation on the left anterior dorsum of the tongue in a pediatric patient [39].

Findings from the literature review support this pattern, with approximately half of the reported cases occurring on the gingiva and a smaller proportion involving extragingival sites such as the tongue, lips, and palate [3]. These atypical presentations may pose diagnostic challenges, particularly in children, where PG is less frequently reported [3, 9].

Clinically, PG typically presents as a painless, exophytic lesion with a sessile or pedunculated base and a tendency to ulcerate and bleed due to its high vascularity. In the present case, the history of local trauma and the progressive growth pattern were consistent with previously reported findings, reinforcing trauma as a key triggering factor [1, 3].

The differential diagnosis of PG includes several reactive and neoplastic lesions with similar clinical features, such as hemangioma, peripheral giant cell granuloma, and fibrous epulis [1, 3, 4]. As clinical characteristics alone are insufficient for a definitive diagnosis, histopathological examination remains essential [3]. Consistent with the literature, the diagnosis in this case was confirmed histologically.

Surgical excision remains the most reported treatment, with low recurrence rates when the lesion is completely removed and local irritants are eliminated [1, 4, 9]. In the present case, conventional excision resulted in no short‐term recurrence. Alternative approaches, including laser surgery and electrocautery, have been documented and may provide benefits such as decreased intraoperative bleeding and enhanced patient comfort [2, 8, 15, 16, 34].

In parallel, topical beta‐adrenergic antagonists have emerged over the last decade as a noninvasive therapeutic. A recent systematic review confirms that topical timolol can induce complete resolution in a relevant rate of PGs. However, the available primary evidence is limited. Most data are derived from small, heterogeneous case reports and case series, along with a single comparative clinical study [43].

## 5. Conclusions

This case highlights an uncommon extragingival presentation of PG in a pediatric patient. Although PG most frequently affects the gingiva, clinicians should be aware that it may occur at atypical intraoral sites, such as the tongue, and may mimic other lesions with similar clinical features.

Histopathological examination remains essential for definitive diagnosis, and conventional surgical excision is a reliable and effective treatment approach that yields favorable outcomes and low recurrence when combined with removal of local irritants.

## Funding

This research received no specific grant from any funding agency in the public, commercial, or not‐for‐profit sectors. The study was conducted as part of the academic activities of the Pontifical Catholic University of Rio Grande do Sul (PUCRS). The institution had no role in the study design, data collection, data analysis, manuscript preparation, decision to publish, or the submission of the manuscript.

## Consent

Written informed consent for publication of the clinical information and images included in this case report was obtained from the patient’s legal guardian.

## Conflicts of Interest

The authors declare no conflicts of interest.

## Data Availability

The data supporting the findings of this study are available from the corresponding author upon reasonable request.
